# Where on the rainbow we stand

**DOI:** 10.1017/S1478951526101734

**Published:** 2026-01-29

**Authors:** Zhaohui Su

**Affiliations:** 1 School of Public Health, Southeast University, Nanjing, China; 2 Key Laboratory of Environmental Medicine Engineering, Ministry of Education, School of Public Health, Southeast University, Nanjing, China

Death, after all, is but a goodbye –
A promise life made,
A contract the soul signed –
We meet, we greet,
We miss, we cherish,
We search, we regret,
And then, we say goodbye.

The moments are what matter –
Albeit sometimes carried clumsily,
Albeit often with anxious heartbeats;
The memories are what count –
Albeit sometimes as bitter medicine to swallow,
Albeit often with rambling sighs to utter.
We never knew what might happen,
Between one storm and the next,
But we lived nonetheless,
Speaking of hope as if it were a covenant kept.

The rituals and celebrations,
The loose ends in need of tying up;
The pills and bills,
The unintended consequences that cannot be reversed;
The wills and will-nots,
The bucket lists that must be crossed off –
Sometimes we are too easily moved,
By the drama of our age;
Sometimes we are too calm to be unmoored,
By the irreversibility of our page.

Yet we seldom fixate on time machines.
We are, after all, our own fate-makers –
Builders, dismantlers,
Doubters, and re-creators;
Wreckers, healers,
Seekers, then finders.

Before we ever ask for directions,
For the past or the future entwined,
Ask where on the rainbow we stand right now,
In time’s gentle and mystic design.

## Data Availability

Data are available upon reasonable request.

